# Ivermectin-Induced Apoptotic Cell Death in Human SH-SY5Y Cells Involves the Activation of Oxidative Stress and Mitochondrial Pathway and Akt/mTOR-Pathway-Mediated Autophagy

**DOI:** 10.3390/antiox11050908

**Published:** 2022-05-05

**Authors:** Yuan Zhang, Tun Sun, Meng Li, Yanling Lin, Yue Liu, Shusheng Tang, Chongshan Dai

**Affiliations:** 1 College of Veterinary Medicine, China Agricultural University, No. 2 Yuanmingyuan West Road, Beijing 100193, China; zhangyuan@cau.edu.cn (Y.Z.); suntun@cau.edu.cn (T.S.); s20193050723@cau.edu.cn (M.L.); s20213050778@cau.edu.cn (Y.L.); s20203050760@cau.edu.cn (Y.L.); 2 Beijing Key Laboratory of Detection Technology for Animal-Derived Food Safety, College of Veterinary Medicine, China Agricultural University, Beijing 100193, China; 3 Key Biology Laboratory of Chinese Veterinary Medicine, Ministry of Agriculture and Rural Affairs, Beijing 100193, China

**Keywords:** ivermectin, neurotoxicity, oxidative stress, apoptosis, autophagy, Akt/mTOR pathway

## Abstract

Ivermectin (IVM) could cause potential neurotoxicity; however, the precise molecular mechanisms remain unclear. This study explores the cytotoxicity of IVM in human neuroblastoma (SH-SY5Y) cells and the underlying molecular mechanisms. The results show that IVM treatment (2.5–15 μM) for 24 h could induce dose-dependent cell death in SH-SY5Y cells. Compared to the control, IVM treatment significantly promoted the production of ROS, mitochondrial dysfunction, and cell apoptosis. IVM treatment also promoted mitophagy and autophagy, which were charactered by the decreased expression of phosphorylation (p)-Akt and p-mTOR proteins, increased expression of LC3II, Beclin1, ATG5, PINK, and Pakin1 proteins and autophagosome formation. N-acetylcysteine treatment significantly inhibited the IVM-induced production of ROS and cell death in SH-SY5Y cells. Autophagy inhibitor (e.g., 3-methyladenine) treatment significantly inhibited IVM-induced autophagy, oxidative stress, and cell apoptosis. Taken together, our results reveal that IVM could induce autophagy and apoptotic cell death in SH-SY5Y cells, which involved the production of ROS, activation of mitochondrial pathway, and inhibition of Akt/mTOR pathway. Autophagy inhibition improved IVM-induced oxidative stress and apoptotic cell death in SH-SY5Y cells. This current study provides new insights into understanding the molecular mechanism of IVM-induced neurotoxicity and facilitates the discovery of potential neuroprotective agents.

## 1. Introduction

Ivermectin (IVM), one member of macrocyclic lactone family, has been used as an antiparasitic agent to prevent or treat infections caused by gastrointestinal lungworms, roundworms, and mites in human and veterinary medicine worldwide [1,2]. In general, IVM is a highly selective positive allosteric modulator of glutamate-gated chloride channels in nematodes and insects, and its insecticidal dose has no marked effect in mammalians’ nervous system [3]. The higher concentrations of IVM exhibited antiviral, antimalarial, antimetabolic, and anticancer effects; however, the potential cytotoxicity and toxic effects in humans or animals also increased [3,4,5]. In humans, IVM treatment could induce potential neurotoxicity, gastrointestinal toxicity, and cardiovascular adverse effects in patients. Symptoms were gastrointestinal distress, confusion, ataxia, weakness, hypotension, and seizures [6]. IVM use could also cause neurotoxicity in cats, pigs, sheep, and horses and the clinical symptoms include depression and coma [7,8,9]. In a rat model, the intravenous administration of IVM at the doses of 10 and 15 mg/kg could cause marked neurotoxicity [7]. Tawfeek et al. reported that IVM could cause marked nephrotoxicity in rats via the activation of oxidative stress and mitochondrial apoptotic pathway [10]. Serious neurological adverse events have been reported from large scale community-based IVM treatment campaigns against onchocerciasis volvulus in Africa [11]. Recently, IVM-caused neurotoxicity in human has raised concerns when it was used to prevent Coronavirus Disease 2019 (COVID-19) [6]. To date, there is little knowledge on IVM-caused neurotoxicity in human and animals, and the precise molecular mechanism is largely unknown.

A previous study reported that IVM has a higher affinity to p-glycoprotein (p-gp) and crosses the blood–brain barrier into the brain tissues in mammal animals with difficulty [1]. The increased incidences of IVM-mediated neurotoxicity had been detected in some animals or human populations who had the reduced expression or functional loss of p-gp in brain tissues [12,13]. Previous studies have demonstrated thar IVM-mediated cytotoxicity involved various pathways, including mitochondrial pathway, cell cycle, oxidative stress, AMP-activated protein kinase (AMPK) pathway, and mitogen-activated protein kinases (MAPKs) pathway [14,15]. Very recently, Zhang et al. reported that IVM could induce an inflammation response on macrophages via the activation of nuclear factor kappa light chain enhancer of activated B cells (NF-κB) signaling pathway [16]. IVM could also induce cell autophagy in several cancer cells, including human breast cancer cells, SK-MEL-28, glioma U251 and C6 cells, and HeLa cells [4,17,18,19]. A previous study reported that IVM at a lower dose (i.e., 1 or 3 μM) could not only increase the drug sensitivity of tumor cells, but also reverse drug resistance by inhibiting the EGFR/ERK/Akt/NF-κB pathway in human HCT-8 colorectal cancer cells [20]. It was also demonstrated that IVM could directly interact with the extracellular domain of EGFR, which plays an important role in neuronal cell survival [20,21]. Autophagy is a fundamental cellular process that eliminates molecules and subcellular elements, including damaged proteins, lipids, and nucleic acids, via the lysosome-mediated degradation pathway [22]. In the current study, the potential neurotoxicity of IVM is assessed by using a human neuroblastoma SH-SY5Y cell line, a thrice cloned subline of the neuroblastoma cell line SK-N-SH, which had been used to study the neurotoxicity caused by many drugs or environmental toxins [23,24,25,26]. Importantly, the involvements of apoptosis and autophagy regulation and the potential cross-talk during IVM-induced cell death are further investigated.

## 2. Materials and Methods

### 2.1. Chemicals and Regents

Ivermectin (purity > 97%) was purchased from Hebei Weiyuan Biochemical Co., Ltd. (Shijiazhuang, China). Dulbecco’s modified eagle’s medium (DMEM) and fetal bovine serum (FBS) were both purchased from Gibco BRL (Grand Island, NY, USA). Cell counting kit-8 (CCK-8) was purchased from MedChem Express (Shanghai, China). Dimethyl sulfoxide (DMSO), N-acetylcysteine (NAC), phenylmethylsulfonyl fluoride (PMSF), 3-methyladenine (3-MA), 2′,7′-dichlorfluorescein-diacetate (DCFH-DA), JC-1 mitochondrial detection kit, caspase pan inhibitor Z-VAD-FMK, and Hoechst 33342 kit were all purchased from Beyotime Biotechnology (Shanghai, China). Acridine orange (AO) and chloroquine (purity > 99%) were both purchased from Aladdin (Shanghai, China). All other regents in the present study were of analytical grade.

### 2.2. Cell Cultures

Human neuroblastoma SH-SY5Y cell line was purchased from American Type Culture Collection (CRL-2266; Manassas, VA, USA). Cells were cultured in DMEM medium with 10% FBS, 100 U/mL penicillin (Beyotime, Shanghai, China), and 100 U/mL streptomycin (Beyotime Biotechnology, Shanghai, China) in a humidified incubator at 37 °C with 5% CO_2_ humidified atmosphere.

### 2.3. Measurement of Cell Viability

Cell viabilities were analyzed by the CCK-8 Assay Kit according to our previous published study [27]. In brief, SH-SY5Y cells were seeded at the density of 1 × 10^4^ cells/well in a 96-well plate. After 16 h, cells were then treated with IVM at the various concentrations (i.e., 2.5, 5, 7.5, 10, and 15 μM, respectively) for additional 24 h. The cells in the control group were treated with the equal volume of vehicle (i.e., 0.2% DMSO). Then, 1 h prior to the assay, CCK-8 reagent (10 μL/well) was added to cells. The absorbance at 450 nm was measured with a microplate reader (Tecan Trading AG, Kanton Zürich, Switzerland) and the values were normalized to the cells in the control group.

To assess the effects of oxidative stress and caspase activations on IVM-induced cytotoxicity, cells were pre-treated with NAC (at 10 mM) or Z-VAD-FMK (at 10 μM) for 1 h, followed by treatment with IVM at 10 μM for additional 24 h; then, cell viabilities were examined using the CCK-8 method.

### 2.4. Measurement of Cell Apoptosis by Flow Cytometer and Hoechst 33342 Staining

Annexin V-FITC/PI Apoptosis Detection Kit (Vazyme Biotech Co., Ltd., Nanjing, China) was used to measure the apoptotic rates by using flow cytometer. The detail protocol was followed according to the previous description [28]. Briefly, SH-SY5Y cells were treated with IVM at the doses of 2.5, 5, 7.5, 10, and 15 μM. After 24 h, cells were washed with PBS, trypsinized with 0.25% EDTA-free pancreatin (Beyotime, Shanghai, China), and harvested. Then, cells were resuspended in 500 μL Annexin V-FITC binding buffer and incubated with 5 μL Annexin V-FITC and 5 μL PI for 15 min in the dark at room temperature. Finally, cell apoptotic rates were measured by flow cytometry (Becton Dickinson, San Jose, CA, USA). All tests were performed in three independent times.

To confirm the induction of apoptotic cell death, deoxynucleotidyl transferase-mediated dUTP nick-end labeling (TUNEL) staining was carried out by using a commercial TUNEL staining kits (Vazyme Biotech Co., Ltd., Nanjing, China). In brief, SH-SY5Y cells were treated with IVM at the dose of 10 μM for 24 h; then, cells were stained according to the instruction’s protocols. Cell nuclei were labelled by 4′-6-diamidino-2-phenylindole (DAPI).

The nuclear morphological changes of IVM-treated SH-SY5Y cells were also observed by using the Hoechst 33342 staining (Beyotime, Shanghai, China) method according to the instructions of the kit. In brief, SH-SY5Y cells were seeded in a 6-well plate and similar dose treatments were performed as described above. Cells were stained with 10 μg/mL Hoechst 33342 dye for 30 min in the dark. Finally, nuclear morphological changes were observed by using a fluorescence microscope (at 340 nm of excitation wavelength and 460 nm of emission wavelength) (Leica Microsystems, Wetzlar, Germany). Apoptotic cells were defined as the increased chromatin condensation and DNA fragmentation.

### 2.5. Measurements of the Production of Reactive Oxygen Species (ROS) and Biomarkers of Oxidative Stress

The levels of cellular ROS were measured by using the fluorescent probe 2,7-dichlorofluorescein diacetate (DCFH-DA) staining method, according to our previous study [28]. In brief, SH-SY5Y cells were seeded at the density of 4 × 10^5^ cells/well in a 6-well plate. After 16 h, cells were treated with IVM at the doses of 2.5, 5, 7.5, 10, and 15 μM. After 24 h, cells were incubated with 2,7-dichlorofluorescein diacetate (DCFH-DA) dye at the final concentration of 10 μM for 20 min at 37 °C in the dark. After three washes with PBS, DCFH-DA fluorescence was observed by using a fluorescence microscope (excitation wavelength: 488 nm; emission wavelength: 525 nm). About 50 cells in each group were randomly selected, and the fluorescence intensity of each cell was analyzed quantitatively using ImageJ software.

The biomarkers of oxidative stress, including the levels of malondialdehyde (MDA), and the activities of superoxide dismutase (SOD) and catalase (CAT), were examined by using commercial MDA, SOD, and CAT kits according to the manufacturer’s instructions (Nanjing Jiancheng Biological Engineering, Nanjing, China), respectively. The protein concentration of each sample was determined by BCA™ Protein Assay Kit (Thermo Fisher Scientific Inc, Dallas, TX, USA). Finally, the levels of MDA, SOD, and CAT of each sample were normalized to protein concentrations.

### 2.6. Measurement of Mitochondrial Membrane Potential

To assess the impact of IVM treatment on mitochondrial function, changes of mitochondrial membrane potential (ΔΨ_m_) were measured by JC-1 staining according to previous studies [29,30]. In brief, SH-SY5Y cells were seeded at the density of 4 × 10^5^ cells/well in a 6-well plate and were treated with IVM at the doses of 2.5, 5 and 10 μM. After 24 h treatment, cells were stained with JC-1 fluorescent probe for 30 min at 37 °C, then washed twice with PBS, and observed using a fluorescence microscope (Leica Microsystems, Wetzlar, Germany). The quantitative analysis of the fluorescence intensity was performed according to our previous study. About 50 cells in each group were randomly selected, and the fluorescence intensity of cells in the green (low membrane potential) and red (high membrane potential) channels were analyzed quantitatively using ImageJ software and their ratios were further calculated.

To further examine the effect of 3-MA treatment on IVM-induced mitochondrial dysfunction, the changes of ΔΨ_m_ were measured by using the Rh123 staining method [29,30]. In brief, SH-SY5Y cells were treated with IVM at 10 μM and co-treated with or without 3-MA at 2 mM for additional 24 h. Cells were stained with Rh123 at the final concentration of 1 μM for 30 min and then washed two times with PBS. Cells were observed by a fluorescence microscopy (excitation wavelength: 488 nm; emission wavelength: 525 nm) and the fluorescence intensity was quantitatively analyzed.

### 2.7. Acridine Orange (AO) Staining

Human SH-SY5Y cells were seeded in 6-well plates and treated with IVM at the final concentrations of 5 and 10 μM for 24 h. Then, cells were incubated with AO at the final concentration of 1 µg/mL for 15 min at 37 °C, followed by washing using PBS for 3 times and acidic vesicular organelles were observed by using a fluorescence microscope (excitation wavelength: 488 nm; emission wavelength: 525 nm).

### 2.8. mRFP-GFP-LC3 Plasmid Transfection

Human SH-SY5Y cells were transiently transfected with the mRFP-GFP-LC3 vector and the detail protocol was followed according to our published study [31]. In brief, cells were transfected with LC3-RFP-GFP plasmids using Exfect2000 (Vazyme, Nanjing, China) for 24 h; then, they were treated with IVM at 5 μM for 24 h. LC3 puncta was observed under a LSM 510 Meta Confocal Microscope (Carl Zeiss Micro Imaging).

### 2.9. Western Blotting

Human SH-SY5Y cells were treated with IVM for 24 h; then, they were lysed using RIPA buffer (Beyotime, Shanghai, China) and ultrasonicated by ultrasonic processor (i.e., 3 s and 5 s apart in each cycle for 10 times, power 22 W) (Branson, MO, USA). Cell lysate samples were centrifuged at 14,000× *g* for 15 min at 4 °C and supernatants were collected. Protein contents were analyzed quantitatively by using the BCA™ Protein Assay Kit. Western blotting was carried out according to our previously published protocols [29,30]. In brief, 15 μg protein of each sample was loaded and separated by 8–15% sodium dodecyl sulfate-polyacrylamide gel electrophoresis (SDS-PAGE), and then transferred to a polyvinylidene fluoride (PVDF) membrane. After blocking with 5% skim milk powder for 2 h, the membranes were incubated with the primary antibody overnight at 4 °C. The primary antibodies, including rabbit polyclonal antibody Cleaved Caspase-3 (#9661), Cleaved Caspase-9 (#9509) (1:1000; Cell Signaling Technology, Beverly, MA, USA), PINK1 (#23274) (1:1000; Proteintech, Chicago, USA), and rabbit monoclonal antibodies against Beclin-1 (#3495), p-mTOR (Ser 2448) (#5536), LC3I/II (#12741), PI3K (#4249), β-actin (#4967), Atg5 (#12994) (1:1000; Cell Signaling Technology, Beverly, MA, USA), Caspase-3 (#19677), PARP-1 (#13371) (1:1000; Proteintech, Chicago, USA), Bcl-2 (#AB112), p-Akt (Ser 473) (BBI life Science, Shanghai, China), and mouse monoclonal Parkin (#66674), Bax (#AF0054), and cytochrome C (CytC) (#AC909) (1:1000; Cell Signaling Technology), were employed. Secondary antibodies (1:5000) against mouse or rabbit were used and incubated for 2 h at room temperature. Protein expression was examined by using chemiluminescent (ECL) gel imaging system (Tanon, Shanghai, China). The expression levels of all proteins were normalized to β-actin.

### 2.10. Statistical Analysis

All data in the present study are expressed as the mean ± standard deviation (SD) unless special instructions. Statistical analyses were performed using one-way analysis of variance (ANOVA) for Tukey’s multiple comparisons post hoc test by using GraphPad Prism 9.0 (GraphPad Software, Inc., La Jolla, CA, USA). A *p*-value < 0.05 was considered as statistical significance.

## 3. Results

### 3.1. IVM Induces a Decrease in Cell Viabilities in SH-SY5Y Cells

**Figure 1:**
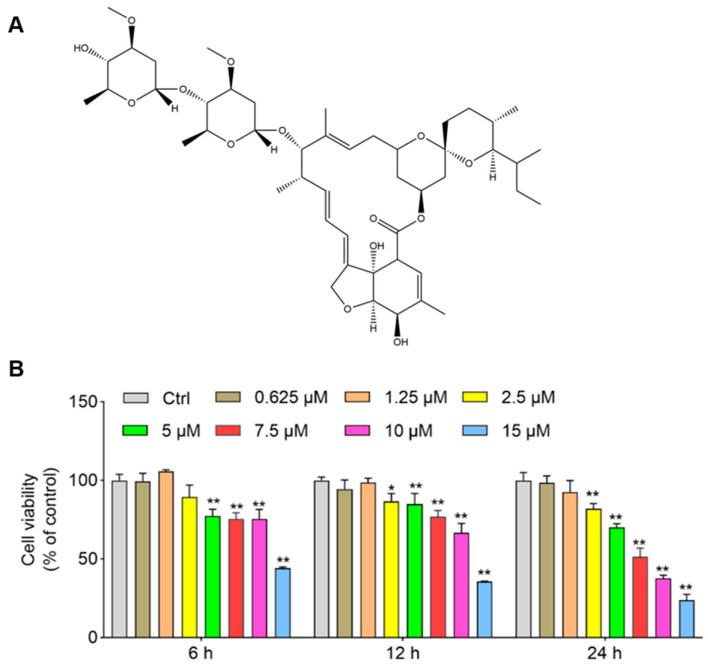
The structure of ivermectin (IVM) and its cytotoxicity in human SH-SY5Y cells. (**A**), the structure of IVM. (**B**), IVM induces cytotoxicity in both time- and dose-dependent manners in human SH-SY5Y cells. Cells were treated with IVM at various doses (i.e., 0.625, 1.25, 2.5, 5, 7.5, 10, and 15 μM) for 6, 12, and 24 h, respectively; the cell viabilities were measured by the CCK-8 method. Data shown are represented as the mean ± SD (*n* = 5); compared to the control group, * *p* < 0.05, ** *p* < 0.01.

As shown in Figure 1, IVM-induced cytotoxicity is dose- and time-dependent. At 6 h and 12 h, IVM treatment at 15 μM significantly decreased the cell viabilities to 44.3% and 35.6% (both *p* < 0.01), respectively; at 24 h, IVM treatment at 0.625, 1.25, 2.5, 5, 7.5, 10, and 15 μM decreased the cell activities to 98.5%, 92.4%, 81.9% (*p* < 0.01), 70.2% (*p* < 0.01), 51.3% (*p* < 0.01), 37.6% (*p* < 0.01), and 23.8% (*p* < 0.01) (Figure 1), respectively. Correspondingly, marked cell morphology changes, including a spindle-like cell body, shrinkage, and dendrite fragmentation in high concentrations of IVM (at 7.5, 10, and 15 μM for 24 h, respectively) were also detected (Supplementary Figure S1).

### 3.2. IVM Induces Apoptotic Cell Death in SH-SY5Y Cells

**Figure 2:**
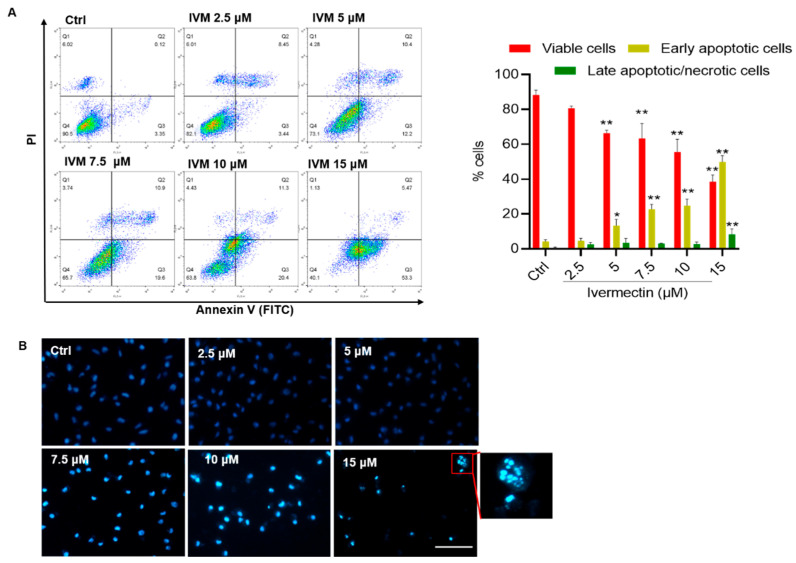
Ivermectin (IVM) induces cell apoptosis in human SH-SY5Y cells. (**A**) apoptotic rates of IVM-treated human SH-SY5Y cells were analyzed by flow cytometry following annexin V-FITV/PI staining. Q1, necrosis cells; Q2, later apoptotic cells; Q3, early apoptotic cells; Q4, live cells. Data shown are represented as the mean ± SD (*n* = 3); compared to the control group, * *p* < 0.05, ** *p* < 0.01. (**B**) The representative images of Hoechst 33342 staining in human SH-SY5Y cells. Nuclear fragmentation (shown in the enlarged image) was detected in the 15 μM IVM treatment group. Bar = 50 μm.

IVM treatment at the doses of 2.5, 5, 7.5, 10, and 15 μM for 24 h significantly increased apoptotic cell death in human SH-SY5Y cells. As shown in Figure 2A, compared to the control group, the rates of early apoptotic cells increased from 4.3% to 13.3%, 22.7%, 24.8%, and 49.8% (all *p* < 0.01) in SH-SY5Y cells treated with IVM at 5, 7.5, 10, and 15 μM, respectively. Correspondingly, the nuclear morphology changes of IVM-treated SH-SY5Y cells were observed. As shown in Figure 2B, IVM treatment at high doses (i.e., 7.5, 10, and 15 μM) could result in marked increased chromosomal aggregation and nuclear fragmentation, compared to that in the control group. Finally, IVM-induced apoptotic cell death was also confirmed by using the TUNEL method (Supplementary Figure S2).

### 3.3. IVM Promotes the Production of ROS and Induces Oxidative Stress in SH-SY5Y Cells

**Figure 3:**
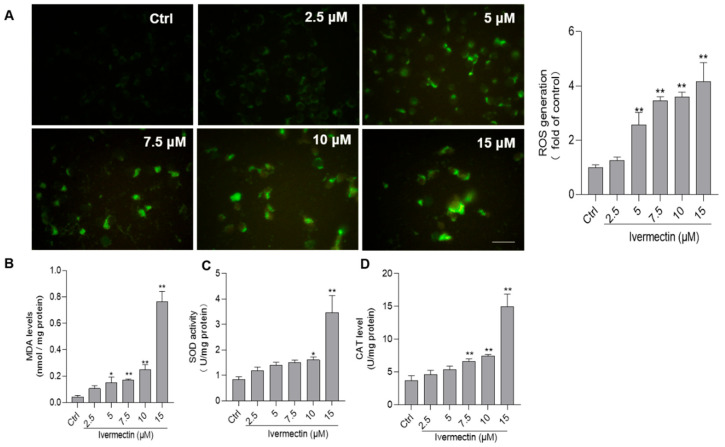
Ivermectin (IVM) induces oxidative stress in human SH-SY5Y cells. (**A**) cells were treated with IVM at the concentrations of 2.5, 5, 7.5,10, and 15 μM for 24 h, then cells were stained the fluorescent probe 2,7-dichlorofluorescein diacetate (DCFH-DA), and the representative images (on the left) were selected and the fluorescence intensities were quantitatively analyzed (on the right). Bar = 25 μm. (**B**–**D**) represent the results of the levels of MDA (**B**) and the activities of SOD (**C**) and CAT (**D**), respectively. All data shown are represented as the mean ± SD, from three independent experiments (*n* = 3); compared to the control group, * *p* < 0.05, ** *p* < 0.01.

Compared to the control, as shown in Figure 3A, IVM treatment at the concentrations of 2.5, 5, 7.5, 10, and 15 μM significantly increased the levels of ROS to 1.3-, 2.6-(*p* < 0.01), 3.4-(*p* < 0.01), 3.6-(*p* < 0.01), and 4.2-fold (*p* < 0.01), respectively. Furthermore, the biomarkers of oxidative stress, including levels of MDA, and activities of SOD and CAT were examined in SH-SY5Y cells. As shown in Figure 3B–D, IVM treatment significantly increased the levels of MDA and consistently upregulated the activities of SOD and CAT in a dependent manner. Compared to the control group, IVM treatment at the doses of 10 and 15 μM significantly increased the levels of MDA to 0.25 nmol/mg and 0.76 nmol/mg protein (both *p* < 0.01) (Figure 3B), respectively; increased the activities of SOD to 1.61 U/mg and 3.48 U/mg protein (both *p* < 0.01) (Figure 3C), respectively; and increased the activities of CAT to 7.4 U/mg and 15.0 U/mg protein (both *p* < 0.01) (Figure 3D), respectively. Furthermore, NAC treatment at 10 mM significantly inhibited the IVM-induced production of ROS, decreases cell viabilities, and caspase-3 activation (Supplementary Figure S3).

### 3.4. IVM Upregulates the Mitochondrial Pathway in SH-SY5Y Cells

**Figure 4:**
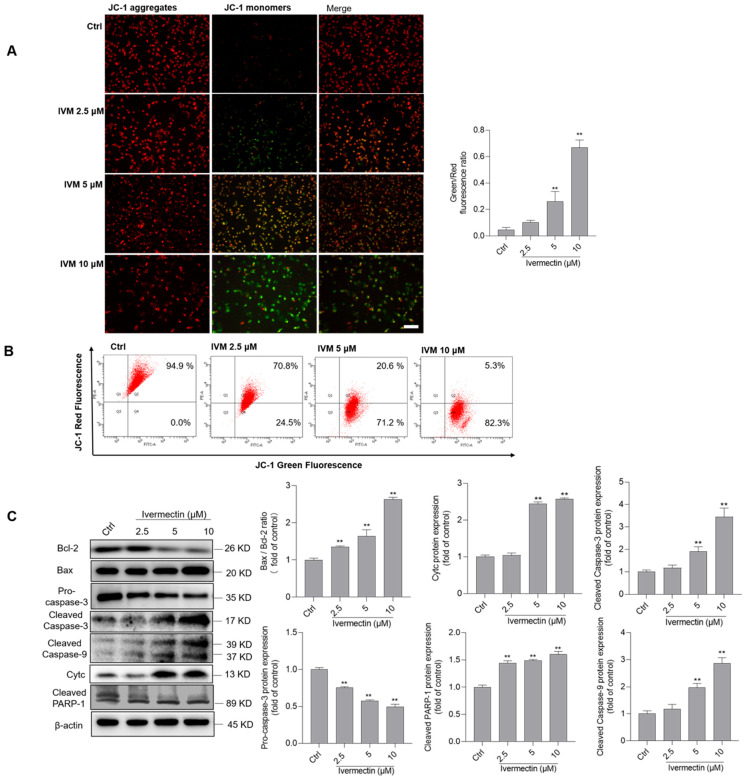
Ivermectin (IVM) induces mitochondrial dysfunction and activates the mitochondrial apoptotic pathway in human SH-SY5Y cells. (**A**) cells were treated with IVM at 2, 5, 5, and 10 μM for 24 h, respectively, and the changes of the mitochondrial membrane potential (ΔΨ_m_) were examined by JC-1 staining. The representative images (on the left) were selected and quantitative analysis (on the right) was performed. Bar = 50 μm. (**B**) the flow cytometry analysis of JC-1 staining. (**C**) the expressions of Bcl-2, Bax, cleaved PARP-1, and CytC proteins were examined by using the Western blotting method. The representative gels (on the left) and quantitative analysis (on the right) are shown. Data shown are represented as the mean ± SD, from three independent experiments (*n* = 3); compared to the control group, ** *p* < 0.01.

As shown in Figure 4A, JC-1 straining showed that IVM treatment significantly decreased the ΔΨ_m_ in a dose-dependent manner. For IVM treatment at 10 μM for 24 h, the fluorescence ratio of green/red increased to about 1.5-fold (*p* < 0.01), compared to that in the control group. Similar results were also confirmed by the flow cytometry analysis (Figure 4B). Furthermore, protein markers of the mitochondrial pathway were examined using Western blotting. As shown in Figure 4C, IVM treatment significantly increased the expressions of Bax, cleaved caspase-3, cleaved caspase-9, cleaved PARP-1 proteins, and CytC proteins, and decreased the expressions of Bcl-2 and pro-caspase-3 proteins. Compared to the control group, IVM treatment at 10 μM significantly increased the ratio of Bax/Bcl-2 to 2.6-fold (*p* < 0.01); increased the expressions of cleaved caspase-3, cleaved caspase-9, cleaved PARP-1, and CytC proteins to 3.5-, 2.9-, 2.6-, and 1.6-fold, respectively (all *p* < 0.01); and decreased the expressions of pro-caspase-3 to 0.49-fold (*p* < 0.01), compared to these in the untreated cells. Furthermore, caspase pan-inhibitor Z-VAD-FMK was used, and it significantly inhibited IVM-induced cytotoxicity and apoptosis (Supplementary Figure S4).

### 3.5. IVM Treatment Activates Cell Autophagy and Inhibits the Akt/mTOR Pathway in Human SH-SY5Y Cells

**Figure 5:**
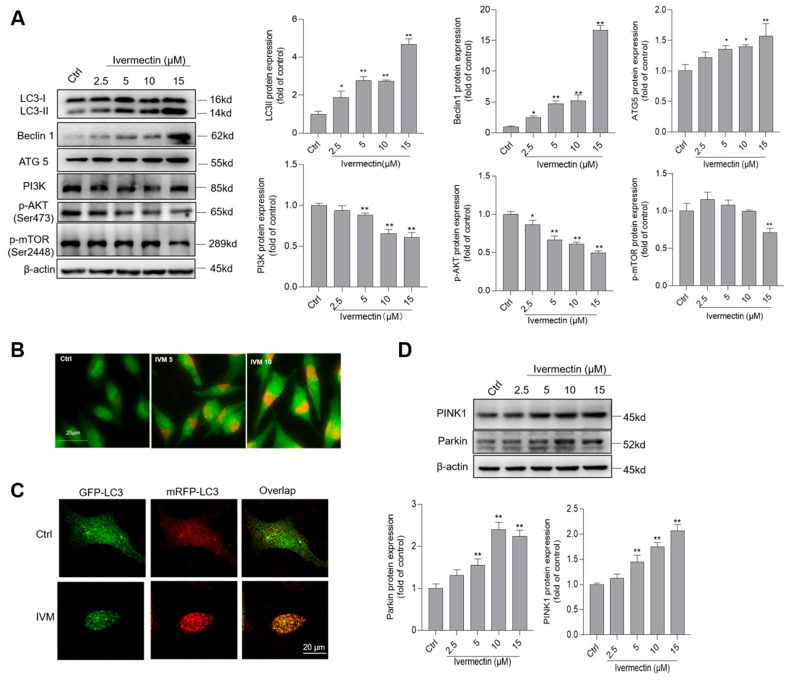
Ivermectin (IVM) induces autophagy and inhibits Akt/mTOR pathways in human SH-SY5Y cells. (**A**) the expressions of LC3II, Belcin1, ATG5, PI3K, p-Akt (ser 473), and p-mTOR (ser 2448) proteins in IVM (at 2.5, 5, 10, and 15 μM for 24 h)-treated cells were examined by using Western blotting. The representative gel (on the left) and quantitative analysis (on the right) are shown. (**B**) acidic vesicular organelles were detected using acridine orange (AO) staining. Bar = 25 μm. (**C**) autophagy flux was monitored by the mRFP-GFP-LC3 plasmid transfection method. Cells were treated with IVM treatment at 5 μM for 24 h, autophagosomes and autolysosome were observed and photographed by a laser scanning confocal microscope. Bar =20 μm. (**D**) IVM treatment activates mitophagy. The expressions of PINK1 and Parkin proteins were examined. Data shown are represented as the mean ± SD, from three independent experiments (*n* = 3); compared to the control group, * *p* < 0.05, ** *p* < 0.01. CQ 5, chloroquine 5 μM; CQ 10, chloroquine 10 μM.

As shown in Figure 5A, IVM treatment at 2.5, 5, 10, and 15 μM significantly increased the expressions of LC3II, Beclin1, and ATG5 proteins, compared to these in the control group. When the cells were treated with IVM at 15 μM for 24 h, the expressions of LC3II, Beclin1, and ATG5 proteins increased to 4.7-, 16.7-, and 1.6-fold, respectively (all *p* < 0.01), compared to the corresponding control. In addition, significantly downregulated expressions of PI3K, p-Akt, and p-mTOR proteins in IVM-treated cells were detected (Figure 5A). We also performed the autophagy observation by using AO staining. As shown in Figure 5B, IVM treatment at 5 and 10 μM for 24 h significantly increased the levels of cytoplasmic acidic vesicular organelles, compared to the control group. Moreover, autophagy flux in IVM-treated cells was further assessed by using mRFP-GFP-LC3 transfection. As shown in Figure 5C, SH-SY5Y cells were treated with IVM at 5 μM for 24 h; the increased formation of autophagosomes and autolysosomes that are evidenced by the yellow and red puncta, respectively, are detected, indicating that IVM activated autophagy and upregulated autophagy flux in SH-SY5Y cells. We further examined the expression of PINK1 and Parkin proteins, two biomarkers of mitophagy. For the IVM 15 μM group, the expression of PINK1 and Parkin proteins increased to 2.1- and 2.3-fold, respectively, compared to the control group (Figure 5D).

### 3.6. Inhibition of Autophagy Improves Ivermectin-Induced Cytotoxicity, Oxidative Stress, and Apoptotic Cell Death

**Figure 6:**
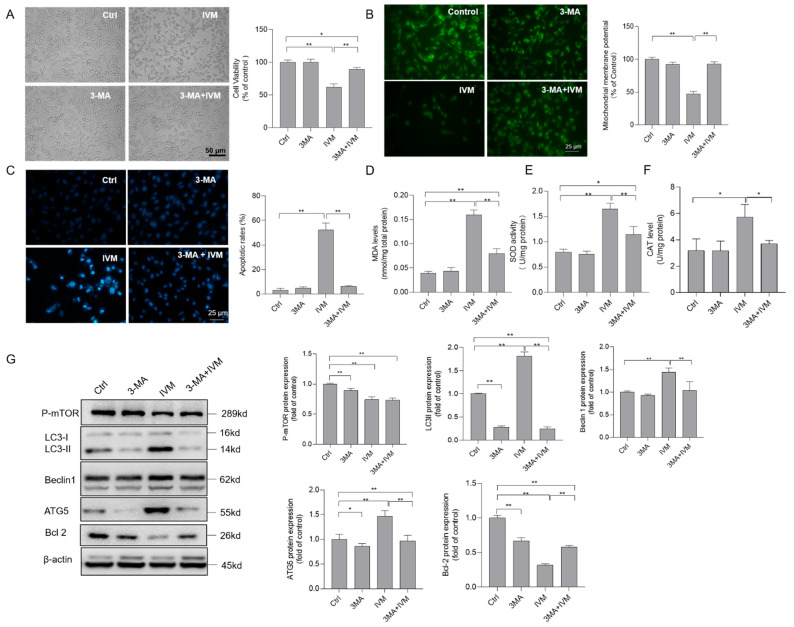
Inhibition of autophagy decreases ivermectin (IVM)-induced cytotoxicity, oxidative stress, and the mitochondrial apoptotic pathway in human SH-SY5Y cells. (**A**) cells were treated with IVM at 7.5 μM with or without 3-methyladenine (3-MA) (2 mM) for 24 h, changes in the cell morphology were observed, and cell viability was examined by the CCK-8 method. Bar = 50 μm. (**B**) changes in the mitochondrial membrane potential were examined using Rh123 staining. The representative gel (on the left) and quantitative analysis (on the right) are shown. Bar = 25 μm. (**C**) cell apoptotic rates were analyzed by using the Hoechst 33342 staining method. The representative gel (on the left) and quantitative analysis (on the right) are shown. Bar = 25 μm. (**D**–**F**) biomarkers of oxidative stress, including levels of MDA (**D**), activities of SOD (**E**), and CAT (**F**), respectively, were measured. (**G**) The expressions of autophagy-related proteins, including p-mTOR, LC3II, Beclin1, ATG5, and Bcl-2 proteins, were examined by Western blotting. Data shown are represented as the mean ± SD, from three independent experiments (*n* = 3); * *p* < 0.05, ** *p* < 0.01.

In the present study, according to the results obtained for cell viability, IVM dose at 7.5 μM was selected to investigate the role of autophagy inhibitor 3-MA on IVM cytotoxicity. As shown in Figure 6A, 3-MA treatment at 2 mM significantly inhibited IVM (at 7.5 μM for 24 h)-induced cytotoxicity and decreased the cell viabilities in SH-SY5Y cells. Meanwhile, 3-MA treatment significantly abolished IVM-induced cytotoxicity and the loss of ΔΨ_m_ (Figure 6B) and cell apoptosis (Figure 6C), compared to treatment with IVM alone. Furthermore, 3-MA treatment significantly inhibited the increase in MDA levels, and the decreases in SOD and CAT activities caused by IVM (Figure 6D–F), indicating that 3-MA treatment could block IVM-induced oxidative stress damage. The expressions of protein related with apoptosis and autophagy were further examined. As shown in Figure 6G, 3-MA treatment did not change the expression of p-mTOR (ser 2448), but significantly decreased the expressions of Beclin1, ATG5, and LC3 proteins (all *p* < 0.01), respectively, and increased the expression of the Bcl-2 protein (*p* < 0.01), compared to treatment with IVM alone. There was no marked cell cytotoxicity in the 3-MA alone treatment group, compared to that in the untreated cells.

## 4. Discussion

IVM has been widely used as an antiparasitic drug in human and veterinary medicines [1]. Moreover, recent studies showed that IVM exhibited several new threptic effects, including anti-cancer, anti-inflammation, anti-diabetic, and antiviral effects [3,32]. Specifically, the in vitro study showed that the higher dose of IVM could inhibit the virus replication of severe acute respiratory syndrome coronavirus 2 (SARS-CoV-2). However, it is controversial in clinical practice due to its toxic effects [3,33]. In relation to the current recommended dose, IVM is not thought to readily cross the blood–brain barrier in humans as it is excluded by the p-gp drug pump. It has been reported that an IVM overdose could induce neurotoxicity in SARS-CoV-2 patients and the main neurotoxic symptoms include confusion, ataxia, weakness, hypotension, and seizures [6]. In another study, there was a total of 426 reports concerning 1,668 people receiving an oral administration of IVM exhibiting neurological adverse events, and the main symptoms included pruritus (25.3%), headache (13.9%), and dizziness (7.5%). Animal experiments showed that the intravenous administration of IVM at the doses of 10 or 15 mg/kg in rats could cause marked central nervous system (CNS) depression [7]. Unfortunately, to date, there is very limited treatment and prevention strategies for IVM-induced neurotoxicity in humans and animals. Therefore, it is urgent to investigate the molecular mechanism of IVM-induced neurotoxicity.

The previous studies showed that IVM could induce marked cytotoxicity and cell apoptosis in various cell lines, including human MCF-7 breast cancer cells, colorectal cancer SW480 and SW1116 cells, K562 chronic myeloid leukemia cells, mouse Raw264.7 cells, HeLa cells, bovine mammary gland epithelial cells, porcine trophectoderm (pTr) and uterine luminal epithelial (pLE) cells, and Chinese hamster ovary (CHO) cells [4,14,16,19,34,35,36,37]. In the current study, our results show that, for the first time, IVM treatment at the doses of 2.5–15 μM for 24 h could cause marked cytotoxicity and apoptotic cell death in human SH-SY5Y cells (Figure 1 and Figure 2). The cytotoxicity reported in this present study is similar to these previous studies. Furthermore, our current data that presents that IVM could induce apoptotic cell death in human SH-SY5Y neuronal cells by using various apoptosis detection methods, including TUNEL, Hoechst 33342 staining, Annexin V-FITC/PI, and cleaved caspase-3 protein expression (Figure 2 and Figure 4, and Supplementary Figure S2). Furthermore, our study demonstrated that IVM-induced apoptosis involved the production of ROS, oxidative stress, mitochondrial dysfunction, autophagy activation, and Akt/mTOR pathway (Figure 1, Figure 2, Figure 3, Figure 4, Figure 5 and Figure 6).

The excessive production of ROS could result in oxidative stress and lead to damaging effects on lipids, proteins, and DNA, and ultimately cell death [29]. ROS commonly consists of hydrogen peroxide (H_2_O_2_), superoxide radical anion, and the hydroxyl radical [38]. Usually, the nervous system has the oxygen demand and high polyunsaturated fatty acid content and is highly sensitive to oxidative damage [38]. Many studies reported that oxidative stress plays critical roles in various environment toxins or antibiotic drugs-induced neurotoxicity, such as the T-2 toxin, cadmium, and colistin [29,39,40]. Previous studies reported that IVM exposure could result in oxidative stress in human HeLa cells and K562 cells [19,36]. Consistently, in the present study, IVM treatment at 2.5–15 μM for 24 h significantly increased the production of ROS, MDA levels, and SOD and CAT activities in human SH-SY5Y cells (Figure 3). MDA is a biomarker of the peroxidation of membrane lipids [41]. SOD and CAT are two important endogenous antioxidant enzymes in response to oxidative stress [42]. Similarly, Ogueji et al. showed that IVM induced liver dysfunction with the significant increases in MDA levels, and the activities of SOD and CAT in *Clarias gariepinus* [43]. The previous study indicated that antioxidant vitamin C could effectively improve IVM-indued nephrotoxicity in rats [10]. In the present study, antioxidant NAC treatment significantly inhibited the IVM-induced production of cellular ROS and cytotoxicity (Supplementary Figure S1), indicating that excessive ROS production plays a critical role in IVM-induced oxidative stress, lipid peroxidation, and cell death.

In mammalian cells, mitochondria are not only the main organelle of ROS, but are also the main target [39]. In vitro studies showed that IVM treatment could cause mitochondrial dysfunction, which is characterized by the decrease in ΔΨ_m_, mitochondrial respiration, and ATP production, and increased mitochondrial ROS production in multiple cell lines [5]. IVM treatment also significantly inhibited the activities of mitochondrial complex I [36]. It was demonstrated that CI- and II-deficient cells displayed an increase in mitochondrial ROS production [44,45]. These evidences indicated that dysfunctional mitochondrial complex I may play a critical role in the IVM-induced production of ROS and mitochondrial toxicity. In the present study, our data showed that IVM treatment significantly induced the loss of ΔΨ_m_ and increased the ratio of Bax/Bcl-2 in human SH-SY5Y cells (Figure 4A,B). It is known that the increased ratio of Bax/Bcl-2 could trigger the opening of the mitochondrial permeability transition pore, which, together with the disrupted ΔΨ_m_, triggered the releases of CytC, cascading to the activation of caspases (e.g., caspases-9 and -3), finally resulting in cell apoptotic death [46]. Caspase-3 and cleaved PARP-1 are two important apoptotic markers [30,42]. Caspase-9 is a biomarker of the mitochondrial apoptotic pathway [47]. In the present study, our data showed that IVM treatment could significantly increase the expression of cleaved caspase-9, cleaved caspase-3, and cleaved PARP-1 protein expression (Figure 4). Meanwhile, caspase pan-inhibitor Z-VAD-FMK treatment could significantly inhibit IVM-induced cytotoxicity and apoptosis (Supplementary Figure S4). Taken together, this evidence indicates that the mitochondrial apoptotic pathway plays a critical role in IVM-induced cytotoxicity and neurotoxicity.

Autophagy is a lysosome-dependent intracellular catabolic process [48]. In general, the activation of autophagy may provide energy and material by self-digestion for macromolecular biosynthesis and promotes the survival from starvation [48,49]. In the present study, (i). the IVM induction of autophagosome formation was demonstrated by the immunodetection of LC3II, Beclin1, and ATG5 (Figure 5A). LC3II and Beclin1 are markers of autophagy and they mediate the initiation and closure of the autophagic vesicle, respectively [31,50]. ATG5 is a key gene in regulating autophagosome formation and expansion [48]. (ii). IVM treatment increased lysosomal acidification (Figure 5B). (iii). mRFP-GFP-LC3-transfected SH-SY5Y cells showed that IVM treatment increased the formation of autophagosomes and autolysosomes and increased autophagy flux (Figure 5C). Taken together, this evidence demonstrates that IVM treatment could promote cell autophagy activation. mTORC1 is a regulatory-associated complex responsible for regulating cell growth and proliferation in response to nutrients, such as oxygen, amino acids, and cellular energy levels [51]. mTORC1 also plays a crucial role in regulating autophagy [52]. It can directly interact with the ULK1/2-Atg13-FIP200 complex and regulate ULK1 activity and control autophagy [51]. Therefore, as an upstream regulator of mTOR, Akt is usually considered to be an autophagy suppressor [52]. In turn, the activation of Akt is controlled by a multi-step process that involves PI3K [53]. The PI3K/Akt/mTOR pathway has been demonstrated to participate in many neurotic drugs or toxins-regulated autophagy responses, such as colistin, methamphetamine, alcohol, and arsenic [51,54,55]. In the present study, our results show that IVM treatment significantly downregulates the expression of PI3K and its two downstream proteins, p-Akt and p-mTOR (Figure 5A). A previous study reported that IVM treatment at 5–20 μM significantly decreased the expression of p-Akt and p-mTOR in human K562 cells [36]. In another study, it was reported that IVM could activate autophagy by the blockade of the Akt/mTOR signaling pathway in various breast cancer cells. Authors believe that IVM could directly target p21-activated kinase 1 (PAK1), an important interaction protein [4]. Therefore, these data indicate that IVM-induced autophagy in human SH-SY5Y cells is regulated by the inhibition of the Akt/mTOR pathway.

The crosstalk between autophagy and apoptosis was mentioned in many studies [26,28,49,51,52]. Autophagy activation may play a dual role in the process of cell death; this is decided by the drug or cell types [56]. Indeed, IVM-induced autophagy activation has also been demonstrated in human breast cancer cells (i.e., MCF-7 and MDA-MB-231 cells), glioma U251 and C6 cells, HeLa cell, and SK-MEL-28 cells [4,17,18,19]. Interestingly, autophagy activation in these cell lines exhibited functions that were the complete opposite to IVM-induced apoptotic cell death [4,17,18]. In human SK-MEL-28 and glioma U251 and C6 cells, the inhibition of autophagy enhanced IVM-induced apoptotic cell death [17,18]. However, in MCF-7, MDA-MB-231, and HeLa cells, the inhibition of autophagy significantly improved IVM-induced apoptotic cell death [4,19]. In the present study, we found that 3-MA, an autophagy inhibitor that blocks the formation of autophagosomes, significantly attenuates IVM treatment-induced the decrease in cell viabilities, oxidative stress, loss of ΔΨ_m_, and cell apoptosis in SH-SY5Y cells (Figure 6A–F). Our data indicated that autophagy activation caused by IVM could trigger apoptotic cell death in human SH-SY5Y cells. A potential molecular mechanism of autophagy-mediated cell death has been defined, i.e., autophagy induction could promote cell apoptosis by initiating apoptotic signals or degrading apoptotic proteins [56]. A previous study found that ipomoea batatas polysaccharides could induce apoptotic cell death in lung cancer cells via Akt/mTOR-mediated autophagy activation and the autophagic degradation of Bcl-2, a antiapoptotic protein [57]. Similarly, our result shows that autophagy inhibition by 3-MA significantly inhibits the expression of autophagy proteins LC3II, Beclin1, and ATG5, and significantly blocks the decrease in the Bcl-2 protein, then cascades to inhibit mitochondrial dysfunction and apoptosis (Figure 6). Taken together, these data indicate that autophagy activation caused by IVM could trigger the autophagic degradation of Bcl-2, then activate the mitochondrial apoptotic pathway in human SH-SY5Y cells. Notably, although SH-SY5Y cells and glioma U251 and C6 cells are all from the tumor-derived neuronal cell lines [17], IVM-induced autophagy activation played a converse role in cell fate among these different cell lines. This phenomenon indicated that the role of IVM-induced autophagy in cell fate decisions is context dependent. It may also be associated with the differences between 3-MA and CQ, which are both autophagy inhibitors and have different targets [17]. Moreover, our results show that IVM treatment could significantly upregulate the expression of PINK1 and Parkin proteins (Figure 5D), two important biomarkers and functional proteins in the process of mitophagy [58]. Mitophagy is a selective autophagy and it is responsible for eliminating damaged mitochondria to prevent the activation of death signaling. Mitophagy activation usually plays a protective role in cells in response to harmful stresses [59]. Consistently, Jia et al. found that IVM treatment upregulated the expression of PINK1 and Parkin proteins and activated mitophagy in rat H9c2 cells [60]. These data indicated that IVM-activated autophagy may play a dual role, which may be dependent on the activation of mitophagy and anti-apoptotic protein degradation, respectively. The precise molecular mechanisms still need more investigations.

## 5. Conclusions

**Figure 7:**
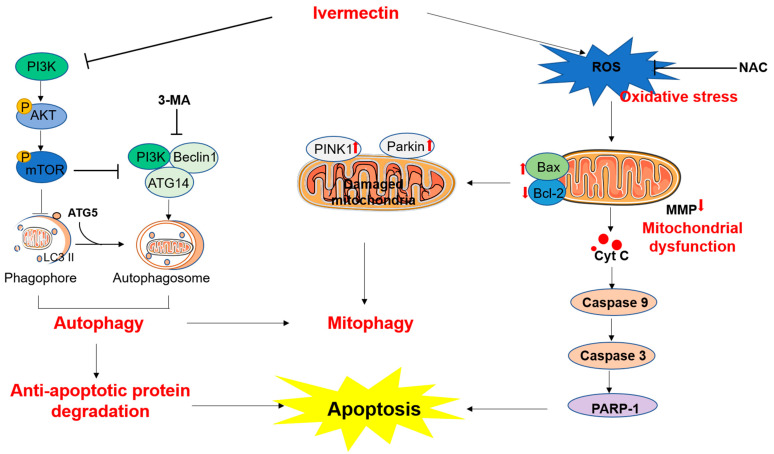
Schematic diagram of the proposed mechanisms of ivermectin-induced apoptosis and neurotoxicity. On the one hand, ivermectin (IVM) treatment upregulated the production of reactive oxygen species (ROS). Excessive ROS could trigger oxidative stress and the mitochondrial apoptotic pathway, finally leading to cell apoptosis. Antioxidant N-acetylcysteine (NAC) supplementation could partly abolish these harmful effects. On the other hand, IVM treatment could downregulate the expression of the PI3K/Akt/mOTR pathway, then induce the formation of autophagosomes and the activation of autophagy. IVM treatment also upregulated the expression of PINK1 and Parkin proteins, and then activated mitophagy. 3-MA could block autophagy and inhibit IVM-induced oxidative stress, mitochondrial dysfunction, and apoptotic cell death.

In conclusion, our results reveal that IVM treatment could upregulate the production of reactive oxygen species (ROS). Excessive ROS could trigger oxidative stress and the mitochondrial apoptotic pathway, finally leading to cell apoptosis (Figure 7). Meanwhile, IVM treatment could downregulate the expression of the PI3K/Akt/mTOR pathway, then induce the formation of autophagosomes and the activation of autophagy (Figure 7). It can also activate mitophagy via the upregulation of PINK1 and Parkin proteins. Moreover, 3-MA could block autophagy and inhibit IVM-induced oxidative stress, mitochondrial dysfunction, and apoptotic cell death in SH-SY5Y cells (Figure 7). Taken together, our current study provides new insights into understanding the molecular mechanism of IVM neurotoxicity and facilitates the discovery of potential neuroprotective agents.

## Data Availability

The data are contained within the article and Supplementary materials.

## Supplementary Materials

The following supporting information can be downloaded at: https://www.mdpi.com/article/10.3390/antiox11050908/s1, Figure S1: Changes of IVM treatment on the cell morphology. Figure S2: Representative TUNEL-stained images. Figure S3: N-acetylcysteine supplementation improves IVM-induced cytotoxicity, ROS production, and caspase activation in human SH-SY5Y cells. Figure S4: Caspase pan-inhibitor Z-VAD-FMK improved IVM-induced cytotoxicity and apoptosis.
