# Supplementary material for: Ivermectin-Induced Apoptotic Cell Death in Human SH-SY5Y Cells Involves the Activation of Oxidative Stress and Mitochondrial Pathway and Akt/mTOR-Pathway-Mediated Autophagy
Source: Antioxidants (Basel). 2022 May 5;11(5):908. doi: 10.3390/antiox11050908 (PMC9137967; doi:10.3390/antiox11050908)
Supplement: Supplementary file 1 [file antioxidants-11-00908-s001.zip › antioxidants-1672574-supplementary-done.pdf]

antioxidants

MDPI

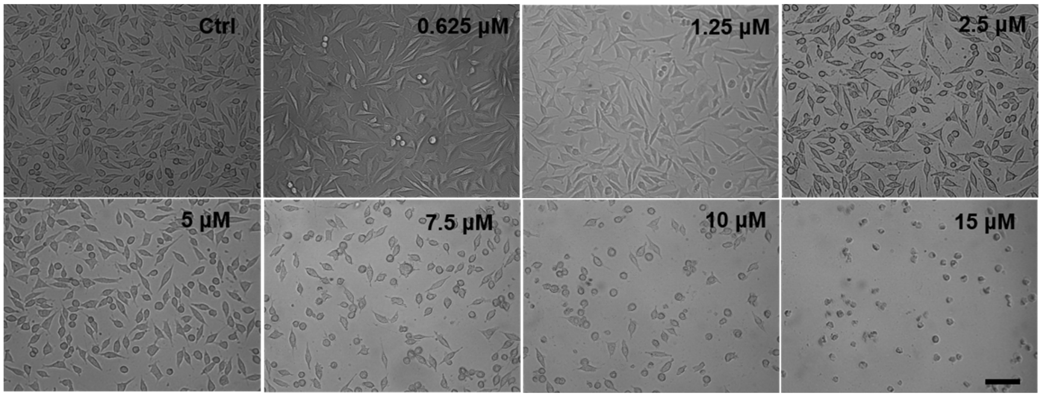

Figure S1. Changes of IVM treatment on the cell morphology. Cells were treated with IVM at the various doses of 0.625, 1.25, 2.5, 5 ,7.5, 10, and 15 μM for 24 h, respectively, the changes of cell morphology were observed. Bar=50 μm.

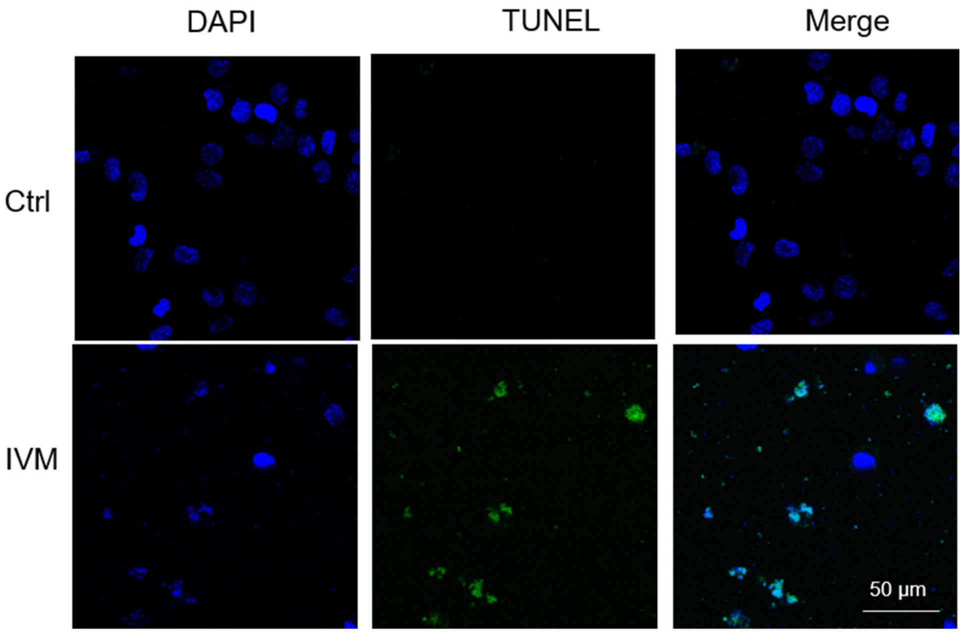

Figure S2. Representative TUNEL-stained images. SH-SY5Y cells were treated with IVM at the dose of 10 μM for 24 h, TUNEL staining were performed according to protocol provided by using a deoxynucleotidyl transferase-mediated dUTP nick-end labelling (TUNEL) kits (Vazyme Biotech Co., Ltd., Nanjing, China). Cell nuclei was labelled by 4′-6-diamidino-2-phenylindole (DAPI). Bar=50 μm.

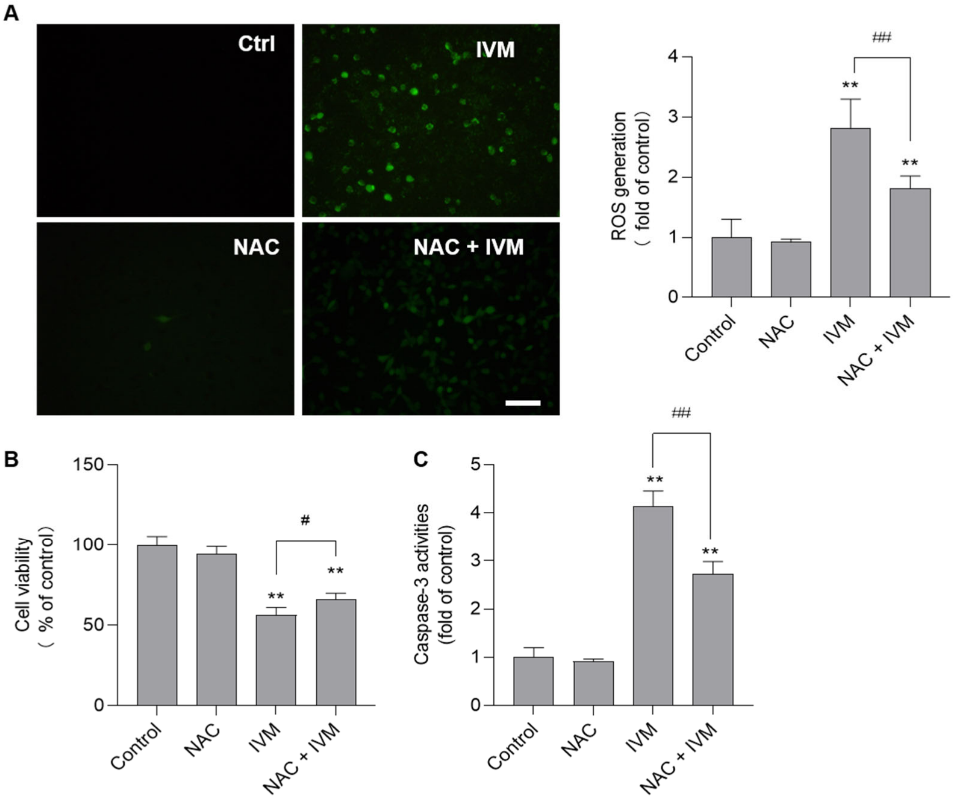

Figure S3. N-acetylcysteine supplementation improves IVM-induced cytotoxicity, ROS production and caspase activation in human SH-SY5Y cells. **A**, Cells were pre-treated with NAC at 10 mM, followed to treat with ivermectin (IVM) at10 μM for 24h, levels of intracellular ROS were stained the fluorescent probe 2,7-dichlorofluorescein diacetate (DCFH-DA), the representative images (on the left) were selected and quantitative analysis (on the right) was performed; Bar = 50 μm. **B**, Cell viabilities were tested by using CCK-8 kit. **C**, The activities of caspase-3 were analysed by using commercial caspase-3 detection kit. The data shown represent the mean ± SD (n=3); Bar=50 μm. Compared to the control group, $^{**}P < 0.01$; Compared to the IVM treatment, $^{\#}P < 0.05$ or $^{\#\#}P < 0.01$.

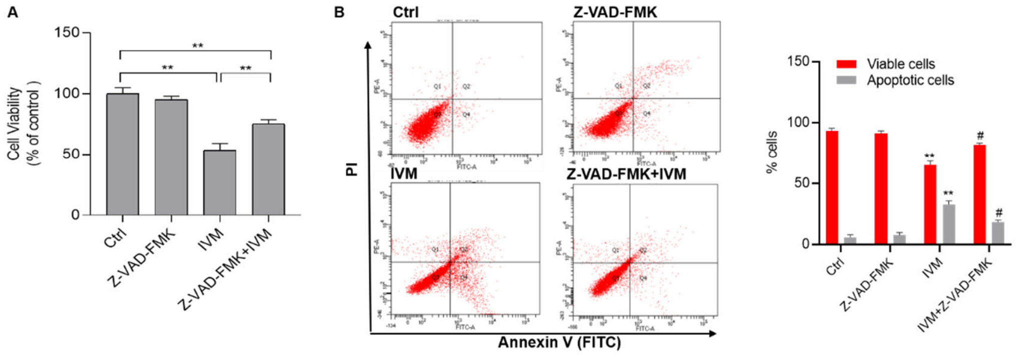

Figure S4. Caspase pan-inhibitor Z-VAD-FMK improved IVM-induced cytotoxicity and apoptosis. Cells were pre-treated with Z-VAD-FMK at 10 μM, followed to treat with ivermectin (IVM) at10 μM for 24 h, then, cell viabilities (**A**) and apoptosis (**B**) were analysed, respectively. Compared to the control group, $^{**}P < 0.01$; Compared to the IVM alone treatment, $^{\#}P < 0.05$.
